# Evaluation of Floxuridine Oligonucleotide Conjugates Carrying Potential Enhancers of Cellular Uptake

**DOI:** 10.3390/ijms22115678

**Published:** 2021-05-26

**Authors:** Anna Aviñó, Anna Clua, Maria José Bleda, Ramon Eritja, Carme Fàbrega

**Affiliations:** 1 Institute for Advanced Chemistry of Catalonia (IQAC-CSIC), Jordi Girona 18-26, E-08034 Barcelona, Spain; anna.clua@iqac.csic.es (A.C.); mjbqbi@cid.csic.es (M.J.B.); carme.fabrega@iqac.csic.es (C.F.); 2 Networking Center on Bioengineering, Biomaterials and Nanomedicine (CIBER-BBN), Jordi Girona 18-26, E-08034 Barcelona, Spain

**Keywords:** 5-fluoro-2′-deoxyuridine, delivery enhancers, cholesterol, palmitic acid, folic acid, GalNAc, conjugation, cancer treatment

## Abstract

Conjugation of small molecules such as lipids or receptor ligands to anti-cancer drugs has been used to improve their pharmacological properties. In this work, we studied the biological effects of several small-molecule enhancers into a short oligonucleotide made of five floxuridine units. Specifically, we studied adding cholesterol, palmitic acid, polyethyleneglycol (PEG 1000), folic acid and triantennary *N*-acetylgalactosamine (GalNAc) as potential enhancers of cellular uptake. As expected, all these molecules increased the internalization efficiency with different degrees depending on the cell line. The conjugates showed antiproliferative activity due to their metabolic activation by nuclease degradation generating floxuridine monophosphate. The cytotoxicity and apoptosis assays showed an increase in the anti-cancer activity of the conjugates related to the floxuridine oligomer, but this effect did not correlate with the internalization results. Palmitic and folic acid conjugates provide the highest antiproliferative activity without having the highest internalization results. On the contrary, cholesterol oligomers that were the best-internalized oligomers had poor antiproliferative activity, even worse than the unmodified floxuridine oligomer. Especially relevant is the effect induced by palmitic and folic acid derivatives generating the most active drugs. These results are of special interest for delivering other therapeutic oligonucleotides.

## 1. Introduction

Antimetabolites are among the oldest but still the most used drugs for chemotherapy of cancer. These include various compounds such as folic acid analogs, purine and pyrimidine analogs, vinca alkaloids, and many antibiotics and other drugs that disturb nucleic acid metabolism and alter DNA synthesis [1]. 5-fluorouracil (FU) and 5-fluoro-2′-deoxyuridine (floxuridine, FdU) are some of the antimetabolites commonly used to treat several cancers, including gastrointestinal, breast and other cancer malignancies [2]. FU and FdU effect as antimetabolites is based on the misincorporation of 5-fluoro-2′-deoxyuridine 5′-triphosphate (FdUTP) into DNA in place of TTP or converted to 5-fluorouridine 5′-triphosphate (FUTP), which competes with UTP during RNA synthesis. Additionally, 5-fluoro-2′-deoxyuridine in his monophosphate form (FdUMP) inhibits thymidylate synthase [3,4], a key enzyme in the synthesis de novo of DNA biosynthesis. DNA modified with FdU has also been shown to be a potent DNA topoisomerases inhibitor [5,6].

Although FU and FdU are clinically effective drugs, side effects and poor oral absorption have been observed during therapy [7]. Several prodrugs of FdU have been explored to improve its physicochemical and bioavailability properties to reduce toxicity [8]. These include alkyl ester, photoactivated, and amino acid ester FdU prodrugs [9,10]. The amino acid ester prodrugs of nucleoside agents have the advantage of being substrates for the PEPT1 peptide transporter [11]. Especially relevant studies have been directed to producing effective 5-fluoro-2′-deoxyuridine phosphoramidate compounds, which can release FdUMP intracellularly [12].

Recently, anti-cancer drugs have been conjugated to nanocarriers with efficient self-assembling properties and drug loading. Following the same approach, some authors explored the conjugation of hydrophobic anti-cancer drugs to short peptides [13] or polyethyleneglycol moieties [14].

In parallel, nucleic acid therapeutics mainly based on antisense and siRNA approaches have been employed for treating several diseases [15]. The latest clinical advances in oligonucleotide therapeutics have demonstrated that the conjugation of agents, such as targeting ligands, aptamers, antibodies or peptides, improve their bioavailability and target tissue exposure of these drugs [16]. Extensive works have been reported claiming the importance of these agents [17,18] in the therapeutic oligonucleotide area. Nowadays, there is extensive research in finding small molecules that recognize cell-membrane receptors for active drug targeting [19]. Particularly relevant results have been obtained by the conjugation of siRNA with GalNAc. This carbohydrate moiety is recognized by the asialoglycoprotein receptor, overexpressed in hepatocytes, improving the selectivity and delivery of nucleic acids to these targeted cells [20].

FdU has also been designed as a building block for the DNA synthesis of therapeutic oligonucleotides for cancer treatment [21]. The authors revealed from preclinical studies that polymeric FdU improved the therapeutic window relative to 5-fluorouracil for several cancer types. The cytotoxicity generated by polymeric FdU is due to the degradation to obtain directly FdUMP that can be used to synthesize FdUTP providing stronger activity than conventional fluoropyrimidine drugs in malignancies for which no chemotherapeutic options provide long term survival.

This approach was also exploited in the synthesis of FdU homopolymers conjugated to lipids [22]. The amphiphilic properties of these conjugates produced efficient polymeric micelles that could interact with serum albumin, facilitating their internalization into cancer cells [23]. Recently, taking advantage of the automated and modular synthesis of therapeutic oligonucleotides based on FdU, we described self-assembled rectangle origami and tetrahedron nanoscaffolds as vehicles for tunable FdU payload in colorectal cancer [24]. Pentameric FdU oligonucleotide was also conjugated to the carrier engineered nanoparticle protein carrying a T22 peptide with an affinity for the CXCR4 chemokine receptor. The conjugates showed selective and potent antitumor activity in the metastatic colorectal cancer model compared to pentameric FdU or FdU alone [25].

Following the concept of these examples, we put forward a new set of promising conjugates based on oligomeric FdU. These polymeric drugs with a well-defined structure and payload are linked to small-molecule enhancers or carriers facilitating the incorporation and drug loading to the cells. We selected lipids, such as cholesterol or palmitic acid and more selective or receptor-mediated molecules, such as GalNAc and folic acid. In addition, polyethyleneglycol (PEG) was also investigated as it is reported that their conjugation improves the in vivo pharmacokinetics [26,27] to reach the targeted cells or tissues [28]. Some of these carriers are more specific for certain cell types; however, the general purpose is to determine the impact of different carriers on therapeutic nucleotides’ delivery and antiproliferative properties.

## 2. Results

### 2.1. Synthesis and Characterization of the FdU_5_ Conjugates

**Scheme 1:**
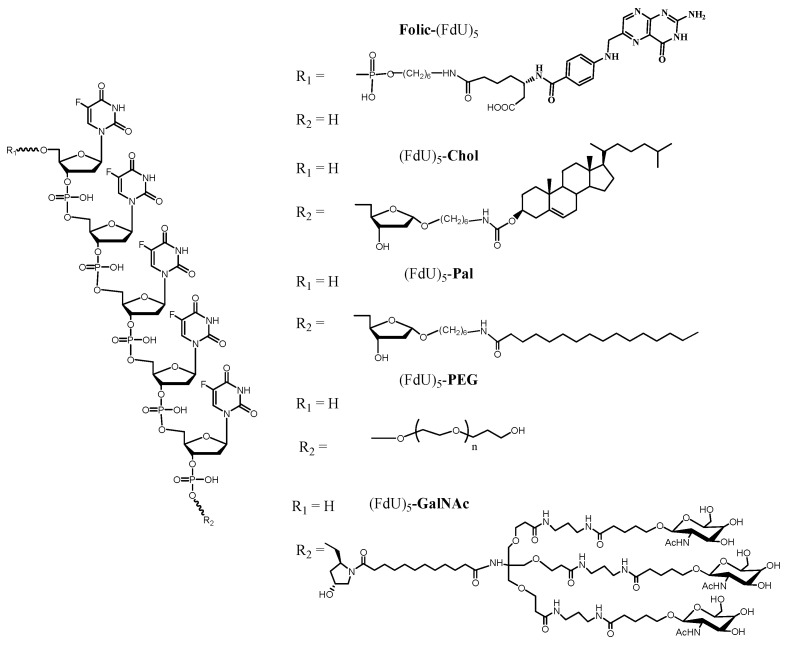
Chemical structure of the pentameric FdU oligonucleotides with the different carriers synthesized in this work.

In this work, we have investigated the effect of several small-molecule internalization enhancers in the antiproliferative and internalization properties of FdU_5_ oligonucleotide. We have prepared and characterized five FdU_5_ conjugates (Scheme 1, Table S1) using solid-phase phosphoramidite chemistry. FdU_5_ linked to cholesterol (FdU_5_-Col), palmitic acid (FdU_5_–Pal) and GalNAc (FdU_5_–GalNAc) were synthesized straightforwardly from the corresponding commercial solid supports functionalized with the appropriate derivatives of the carrier molecules and the phosphoramidite of 5-fluoro-2′-deoxyuridine. For the preparation of the polyethyleneglycol (PEG) FdU conjugate (FdU_5_–PEG), the solid support was prepared following described procedures [29], which can be adapted in principle for any PEG length or shape. In the case of folic conjugated (Folic–FdU_5_), we also prepared the solid support containing FdU [25], and after adding the last FdU phosphoramidite, we incorporated an amino group at the 5′-end of the oligonucleotide using the monomethoxytrityl (MMT)-protected 6-aminohexyl phosphoramidite. Then, solid-phase conjugation with folic acid to the amino-oligonucleotide solid support followed by standard ammonia deprotection produced the desired folic acid conjugate (Folic–FdU_5_).

### 2.2. Enzymatic Degradation of FdU Conjugates

As mentioned above, in this work, the cytotoxicity generated by oligomeric FdU will come from the degradation of the oligomers to produce FdU and FdUMP. Likely, the potential enhancers of cellular uptake introduced at the 3′ or 5′-ends of the oligonucleotides may affect the degradation by exonucleases without preventing this metabolic activation. To confirm this issue, we have analyzed the behavior of the conjugates in the presence of exonucleases followed by reversed-phase analytical HPLC. We have analyzed the degradation of the conjugates using 5′-phosphodiesterase I (snake venom phosphodiesterase, SVP) and 3′- phosphodiesterase II (bovine spleen phosphodiesterase, BSP). Results are shown in Table 1 and Figure S1. As anticipated, unmodified FdU_5_ oligonucleotide was digested in less than one hour using any of both enzymes generating mixtures of FdU and FdU 3′- or 5′-monophosphates that eluted at the beginning of the chromatogram (Figure S1B,C). The degradation of the 3′ conjugates was slower using the 5′-exonuclease (SVP) because this enzyme starts the degradation at the 3′-end that is blocked by the ligands. However, most of the 3′ conjugates were degraded in 24 h except for FdU_5_–GalNAc and FdU_5_–Chol. On the other hand, the degradation of the 5′ conjugate (folic–FdU_5_) by SVP was as fast as the unmodified FdU_5_ as this oligonucleotide has the 3′-end unmodified.

The degradation results by 3′-phosphodiesterase II (BSP) showed in Table 1 demonstrate that most of the conjugates were degraded by BSP except FdU_5_–GalNAc and folic–FdU_5_ that were degraded more slowly than the others. This was expected as BSP starts oligonucleotide degradation by the 5′-end that was unmodified in most of the conjugates except for folic–FdU_5_. We cannot explain the retarded degradation of FdU_5_–GalNAc except for the fact that the GalNAc ligand may be protected by other proteins present in the spleen preparation.

These results agree with the data described by the group of Gmeiner [21], assigning to floxuridine oligonucleotides the role of acting as prodrugs of FdU. The presence of the ligands at the 3′ or 5′-ends may slow the degradation. Nevertheless, they still can act as the metabolic source of the active FdU drug.

### 2.3. Internalization Efficiency of the Different FdU Conjugates

Once the synthesis was completed, internalization experiments were conducted to evaluate the effect of the ligands on the cellular uptake of the conjugates. For this study, the FdU_5_ conjugates were labeled with fluorescein phosphoramidite (FAM) at the 5′-end, except for the Folic–FdU_5_ that the fluorophore was assembled at the 3′-end (Table S1). To this end, a new batch of oligonucleotide conjugates was synthesized with the appropriate solid supports and phosphoramidites to introduce the fluorescein. To prevent the unspecific interaction of the FdU_5_ conjugates with the cellular membrane, the assay was performed by treating the cells with Trypan Blue to quench the extracellular fluorescein and to measure only the intracellular fluorescein signal [30]. Four different cell lines were selected in this study: HeLa (epithelioid cervix carcinoma), HTB-38 and HCC2998 (FU resistant colorectal cancer) and HepG2 (hepatocyte carcinoma).

**Figure 1:**
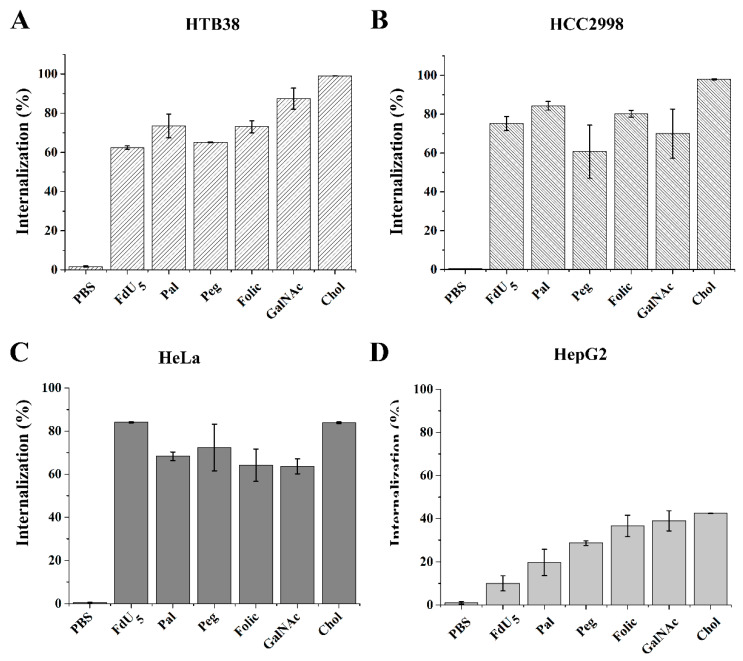
Intracellular uptake of FdU_5_ and FdU_5_ conjugate molecules at 5 µM. The results are presented in bar graphs for the different cell lines (**A**) HTB38 (**B**) HCC2998 (**C**) HeLa, and (**D**) HepG2 cells. Error bars represent the standard deviation (SD) of two independent experiments.

The FdU_5_ oligomer without any modification presented different percentages of internalized oligonucleotide (Figure 1 and Figure S2), reaching the maximum in HeLa cells (84%) and the lowest obtained in HepG2 (10%). The two colorectal cells HTB38 and HCC2998 presented some differences between them, obtaining labeling of 62% and 75% of the cells, respectively. In the case of HeLa cells, the conjugation of FdU_5_ with all the carriers was found to induce a slight reduction in internalization efficacy, except for cholesterol conjugated with a similar level of internalization as found for FdU_5_ alone. HepG2 was found to be the most difficult cell line for gymnotic internalization, but the presence of the ligands improved the internalization of FdU_5_ conjugates. Starting from 10% of labeled cells for the FdU_5_ alone to the maximum percentage of labeled cells obtained with folic acid, GalNAc and cholesterol conjugates (38%, 39% and 42%, respectively). Although these internalization values only arrive near 50% of internalization of the FdU_5_ conjugates in HepG2, the total increase effect produced by the ligands is the highest value comparing with the other cancer lines. The only carrier unable to increase the internalization of FdU_5_ in the two colorectal cancer cells was PEG. The conjugation of FdU_5_ with palmitic or with the folic produced increased the internalization efficiency in both colorectal cancer cell lines of around 15% in HTB38 and 20% in HCC2998. However, cholesterol is the carrier that produced the best internalization values in both cancer cell lines, similar to what was observed for HeLa cells. The FdU_5_–GalNAc was the only conjugate that presented a considerable difference in the internalization effect between the two colorectal cancer HCC2998 (70%) and HTB38 (87%). The internalization increase of GalNAc conjugate in HepG2 cells was expected because of the presence of asialoglycoprotein receptors overexpressed in these cells. However, it is interesting to observe that in the rest of the cancer cell lines, there is also a high internalization of these conjugates. This is probably due to the highly efficient gymnotic uptake of the small FdU_5_ oligonucleotide in these cell lines.

### 2.4. Cytotoxicity Studies of the Different FdU Conjugates

First, we studied the cytotoxic response obtained for FdU_5_ oligomer alone as a control in the four cancer cell lines and wild-type fibroblast, at concentrations ranging from 1 nM to 10 µM (Table 2 and Figure S3) using the 1-(4,5-dimethylthiazol-2-yl)-3,5-diphenylformazan) (MTT) assay. Comparing the MTT results obtained, it is possible to distinguish a different action of FdU_5_ oligomer in each type of cell, being HCC2998 the most resistant with an IC_50_ around 16.9 µM. The cytotoxicity of the FdU_5_ oligomer in HTB38 cells is ten-fold higher, with an IC_50_ of 1.1 µM, demonstrating the different sensitivity to FdU derivatives between these two colon cancer cell lines [31,32]. HepG2 are also fairly resistant to FdU_5_ oligomer with an IC_50_ of 7.8 µM. All three cell lines presented a clear resistance to FdU_5_ cytotoxicity compared to HeLa (IC_50_ 204 nM).

Additionally, we have tested the FdU_5_ derivatives in healthy human fibroblast cells as a model of healthy cells (Table 2, Figures S3 and S8). All the FdU_5_ derivatives were found to induce very low cytotoxicity to this human primary cell culture, as shown in Figures S3 and S8 that include the results of the MTT assays performed in the same range of concentrations used for tumor cells (1 nM to 10 µM). We estimate a CC_50_ value that is shown in Table 2. Due to the low cytotoxicity in healthy cells of all the FdU_5_ conjugates, it was difficult to calculate the selectivity indices (SI = CC_50_/IC_50_). As a broad estimation, the lowest selectivity indices were found for the FdU_5_–PEG conjugate oscillating between 3 and 500 depending on the cell line. For FdU_5_–Pal conjugate, the selectivity indices were between 10 and 1000 and between 13 and 10,000 for FdU_5_–GalNAc conjugate. The selectivity indices for the rest of the derivatives (FdU_5_, FdU_5_–Chol, folic–FdU_5_) were found to be between 10^3^ and 10^5^.

**Figure 2:**
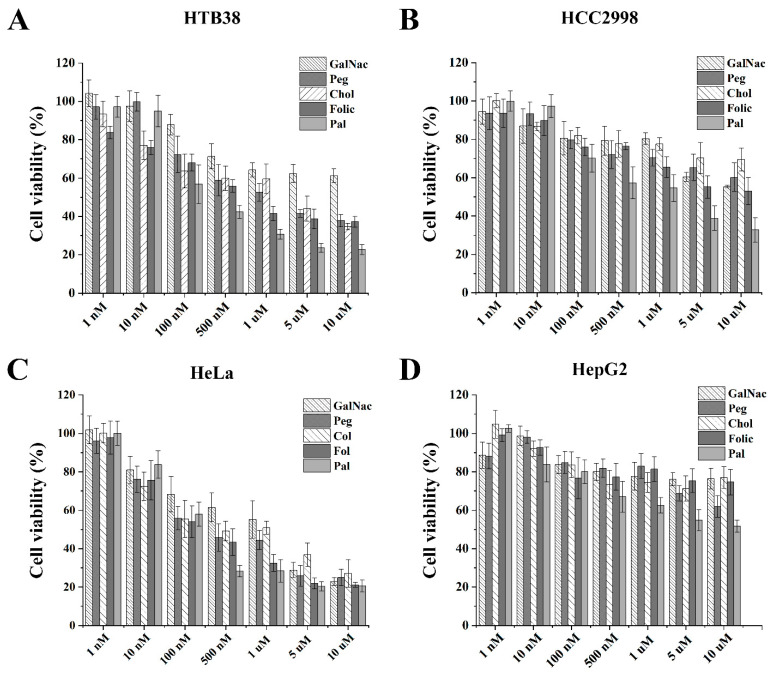
Cytotoxicity activity of the FdU_5_ conjugates in four cancer cell lines. The MTT assays were performed by treating the cells with various concentrations (1 nM to 10 µM) of the oligonucleotide conjugates. Results are presented in bar graphs for the different cell lines (**A**) HTB38 (**B**) HCC2998 (**C**) HeLa, and (**D**) HepG2 cells. Error bars represent standard deviation (SD) of three independent experiments in triplicate.

MTT assays in tumor cells for FdU_5_ conjugated to the internalization elements are shown in Table 2 and Figure 2 and Figures S4–S7. As observed previously with unconjugated FdU_5_, HCC2998 cells presented a higher resistance for all the FdU_5_ conjugates. Only the palmitic acid conjugate and, to less extent, the folic acid conjugate seems to potentiate FdU cytotoxicity having their IC_50_ value in the low micromolar range 1.4 µM and 12.5 µM, respectively. This behavior is also observed in HTB38, where the palmitic conjugate can reduce fourfold the concentration of FdU_5_ to reach the IC_50_ value of 279 nM, and the folic acid conjugate also produced a smaller reduction with an IC_50_ value of 870 nM. Surprisingly, FdU_5_–GalNAc having the best internalization behavior does not correlate with the cytotoxic effect in the HTB38 cells. The slow degradation of these conjugates by phosphodiesterases may explain this low cytotoxic activity. In the case of HeLa cells, only the palmitic conjugate slightly reduces the concentration necessary to reach the IC_50_ (165 nM). The rest of the carriers produced similar or less antiproliferative results than the unconjugated FdU, which could indicate that the maximum cytotoxic effect was already achieved. This is consistent with the internalization results obtained for HeLa cells, where the nanocarriers were ineffective in increasing the internalization of the FdU oligomer.

Surprisingly, cholesterol and pegylated conjugates were less efficient as cytotoxic agents than unconjugated FdU_5_. The reducing FdU antiproliferative effect is probably due to the decreased metabolic generation of the active FdU monophosphate by nuclease degradation, although the slow degradation by phosphodiesterases was only observed for the cholesterol conjugate. Finally, FdU_5_–Pal was ten times more cytotoxic than FdU_5_, in HCC2998 with a selectivity index of around 100 and four times more toxic in HTB38 cells with a selectivity index of around 400. These results are especially relevant as these cells are FU-resistant colorectal cancer cells. Finally, in HeLa cells, FdU_5_–Pal and Folic–FdU_5_ were the more cytotoxic conjugates together with FdU_5_. This is especially relevant as Folic–FdU_5_ and FdU_5_ have very low toxicity in healthy fibroblast cell and consequently the selectivity index is higher than 10^5^.

**Figure 3:**
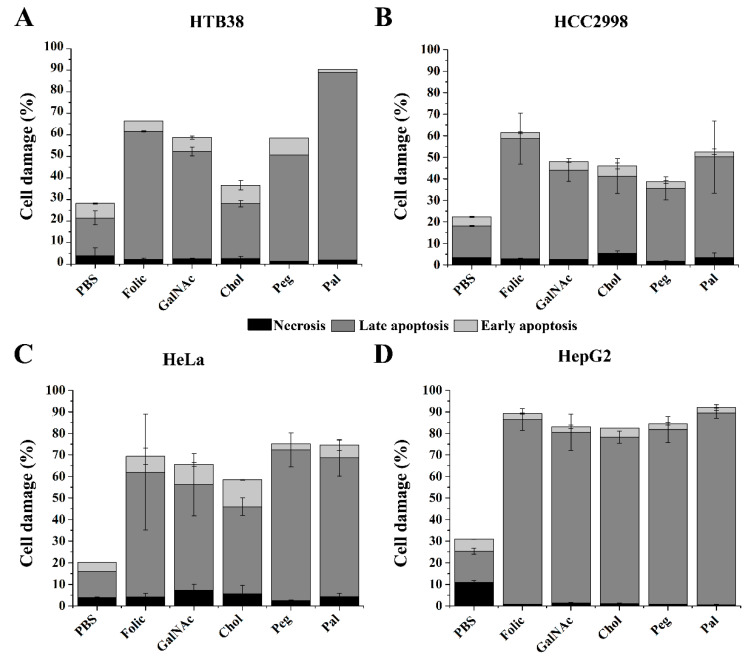
Apoptosis assay comparing the effect produced by the different carriers of FdU_5_ in HTB-38 (**A**), HCC2998 (**B**), HeLa (**C**) and HepG2 (**D**) cells. Error bars represent standard deviation (SD) of two independent experiments.

To compare the different effects on cell death and to identify the cellular death mechanism involved in reducing cell viability of the FdU_5_ conjugates, apoptosis assays were conducted. Overall, we found that all the FdU_5_ conjugates produced a huge increase in late apoptosis, increasing cell death considerably compare with the control in all the cell lines tested (Figure 3). However, we could see different behavior of the FdU nanoconjugates depending on the cell line. In HepG2 cells, no differences in the apoptosis results are observed between ligands. This result is similar to the cytotoxicity results obtained in the MTT assay. In HeLa cells, adding cholesterol generated the less effective conjugate being the PEG and the palmitic ligands the most promising ones. As expected, the two colorectal cancer cells showed different responses to each of the FdU_5_ conjugates being more pronounced in the HTB38. In HTB38, the less effective ligand was the cholesterol conjugate, while in the HCC2998 cell line, PEG conjugate was the less efficient. Clearly, the folic and the palmitic acid ligands are the most promising enhancers to induce apoptosis to cancer cell lines, in agreement with the results obtained in the MTT assay.

## 3. Discussion

We have synthesized and evaluated the antiproliferative and internalizing properties of FdU_5_ conjugates with different ligand molecules demonstrated to facilitate cellular uptake to generate more efficient molecules for cancer therapy. Previous works observed that using pentameric FdU oligonucleotides ligated to a nanoprotein carrier could selectively deliver the FdU drug to metastatic cancer cells improving their pharmacological activity compared to simple FdU [26]. In this case, the conjugation of FdU_5_ to a protein with affinity to CXCR4 receptors overexpressed in cancer stem cells delivered the FdU_5_ oligonucleotide in a very selective and effective way. After this precedent, we were interested in simpler strategies to improve the efficacy of FdU_5_. In the first approach, we studied the conjugation with lipids moieties. The addition of lipids to oligonucleotides has been used for decades to improve the cellular uptake and biodistribution of oligonucleotides and small drug molecules [33]. Cholesterol was one of the pioneering lipophilic modifications introduced in synthetic oligonucleotides, especially in siRNA [34]. Conjugation with cholesterol has demonstrated an important effect on the cellular uptake of therapeutic oligonucleotides, especially in hepatic cells [35]. Palmitic acid has also been introduced in several drugs to enhance delivery to cells. Specifically, it has been introduced at the 5′-end of the sense strand of siRNA, producing an enhancement of the gene-silencing potency due to the nuclease stability and lipophilic character [36].

On the other hand, we considered the attachment of the polymer PEG to the FdU_5_. PEGylation has been used for improving the pharmacokinetic properties of therapeutic drugs. Recent studies have demonstrated that this modification improves cellular uptake, gene regulation efficacy and biodistribution of therapeutic nucleic acids [37].

GalNAc conjugation is the more advanced clinical strategy for therapeutic oligonucleotide conjugates (antisense ODN and siRNA). This ligand mediates binding to the specific surface asialoglycoprotein receptor (ASGR) that is overexpressed on the cell surface of hepatocytes and facilitates productive uptake [38]. The introduction of GalNAc to the nucleoside 5-fluoro 2′-deoxyuridine-5′-phosphate via an amide chain linker was described to deliver the drug to hepatic cells [39]. In this case, the cytotoxicity of FdU oligomers is not improved by the GalNAc conjugation as the drug continues to enter cells by passive diffusion, and no evidence for receptor-mediated effects was observed. However, siRNA GalNAc conjugates are taken by the endocytic pathway as active mediated delivery due to the large macromolecule cargo [40]. Most probably, GalNAc modification will be more efficient in directing the delivery of larger oligonucleotides. For short oligonucleotides, such as FdU small oligomers, the best option has been adding palmitic or folic acid ligands with a clear enhancement of the cytotoxicity in FU-resistant colorectal cancer cells (HTB38 and HCC2998).

Among the conjugates studied in this work, FdU_5_–Pal derivative exhibited the highest antiproliferative activity, and, in addition, it is readily prepared from commercially available reagents or by postsynthetic conjugation of amino-oligonucleotides [41,42]. Palmitic acid–antisense oligonucleotide conjugates were first prepared to increase the binding of antisense oligonucleotides with low-density lipoproteins to improve the leishmanicidal efficiency on intracellular amastigotes [43] and to functionalize cell membranes with oligonucleotides [42]. Soon after discovering the RNA interference, it was shown that siRNAs functionalized with palmitic acid at the 5′-end of the sense strand were substrates for dicer and exhibited significant membrane permeability [36]. Very recently, using palmitic acid derivatives of antisense oligonucleotides have been considered excellent alternatives for extrahepatic delivery of therapeutic oligonucleotides [44,45,46] with enhanced internalization but also enhanced endosomal release [47]. Although all these properties have been found for relatively long oligonucleotides, we could observe some of these excellent properties for short oligonucleotides that must be metabolically activated by nuclease degradation, such as FdU oligomers.

## 4. Materials and Methods

### 4.1. Synthesis and Characterization of FdU_5_ Conjugates

Pentameric FdU conjugates prepared in this study contained a carrier molecule located at the 3′-end except for the folic derivative placed at the 5′-end of the oligonucleotide (Table S1). The conjugates were prepared on a 1 µM scale using commercially available solid supports containing cholesterol, palmitic acid (LGC Link Technologies, Lanarkshire, Scotland) and GalNAc (Primetech ALC, Minsk, Belarus). The synthesis of the folic acid conjugates was done at the 5′-end. In this case, we use solid support functionalized with FdU is synthesized using the corresponding DMT–FdU succinate as described [25]. PEG solid support is prepared from the corresponding succinate derivative of PEG 1000 following the described procedure [29]. The procedure is described in the supplementary section. The addition of the FdU phosphoramidite (LGC Link Technologies, Lanarkshire, Scotland) was done following the standard procedures.

The oligonucleotide carrying folic acid was prepared by solid-phase conjugation of folic acid on pentameric FdU solid support in which an amino group was attached to the 5′-end using the *N*-MMT-6-aminohexyl phosphoramidite. After adding the amino linker, the monomethoxytrityl (MMT) group was removed using the standard detritylation solution. Then, 1.2 mg (2.7 µmols) of folic acid dissolved in 100 μL of anhydrous dimethylformamide (DMF) and activated with PyBOP (benzotriazol-1-yl-oxytripyrrolidinophosphonium hexafluorophosphate), 1.4 mg (2.7 µmols) in the presence of 0.94 µL diisopropylethylamine (DIEA, 5.4 µmols) for 5 min and then reacted with 0.2 µmols of 5′-NH_2_-oligonucleotide solid support for 2 h. Then the solid support was then washed with DMF, acetonitrile (ACN) and dried.

The same oligonucleotide conjugates were prepared for internalization studies as described for the previous sequences using the appropriate fluorescein (FAM) phosphoramidite or the solid support functionalized with fluorescein LGC Link Technologies, Lanarkshire, Scotland) in the opposite end where the carrier molecule was located.

After the assembly of the sequences, the solid supports were treated with aqueous ammonia (32%) at 55 °C for 1 h, filtered, concentrated to dryness and then desalted with a NAP-10 column. For the oligonucleotide with GalNAc derivative, a previous treatment with 0.1 M DBU (1,8-diazabicyclo[5.4.0]undec-7-ene) in ACN for 5 min followed by a 1% Et_3_N wash was used to remove the 2-cyanoethyl protecting group. The oligonucleotides were analyzed by HPLC (supplementary material), and the mass expected was confirmed by MALDI-TOF (Table S1).

### 4.2. Enzymatic Degradation of FdU_5_ and the FdU_5_ Conjugates

Oligonucleotides were subjected to enzymatic degradation followed by HPLC analysis. Briefly, 0.5–1 OD_260_ unit of the oligonucleotide were dissolved in 92 μL of water, 5 μL of 1 M aqueous Tris-HCl solution (pH 8.0) and 1 μL of 1 M MgCl_2_ solution. Then 1 μL of phosphodiesterase I from *Crotalus adamanteus* venom (USB) was added and incubated at 37 °C. Then 30 µL of the solution was removed heated to 85 °C for 3 min. For degradation with phosphodiesterase II, the ODN was dissolved in 100 mM sodium acetate pH 6.5 (100 µL), and 1 µL of phosphodiesterase II from bovine spleens was added. HPLC analysis of the digestion mixture was done using a 15 min linear gradient from 0 to 25% B to analyze the degradation products and then 5 min at 100% B to elute the non-degraded oligonucleotides.

Conditions of the HPLC analysis of the starting oligonucleotides: a 20 min linear gradient from 0 to 50% B except for FU_5_–Pal and FU_5_–Chol that a 20 min linear gradient from 15 to 85% with 10 min at 100% B was used. Column: Nucleosil C18, 10 μm, 250 × 4 mm. Buffer A: 5% acetonitrile (ACN) in 0.1 M triethylammonium acetate (TEAAc) and buffer B: 70% ACN 0.1 M TEAAc.

### 4.3. Internalization by Flow-Cytometry

To assess the internalization level of all the modified FdU_5_ sequences with internalizing elements into these four cancer cells HTB38, HCC2998, HeLa and HepG2, the following procedure was conducted. Cells were seeded in 24-well plates at a density of 8 × 10^4^ cell well^−1^ and incubated for 24 h before treatment. Next, the oligonucleotides were added directly to the cells, dissolved in a fresh growth medium and incubated. Twenty-four hours after sample addition, cells were washed once with phosphate buffer saline (PBS) and harvested by treatment with trypsin.

### 4.4. Cell Proliferation Assay (MTT)

To determine the effect of the FdU_5_ sequences conjugated with internalization elements to the cell proliferation, the method of MTT (1-(4,5-dimethylthiazol-2-yl)-3,5-diphenylformazan) reduction was carried out. Four cell lines, HTB-38, HCC2998, HepG2 and HeLa, were seeded at 5 × 10^4^ cell well^−1^ in 96-wells plates after ensuring that the seeding density was suitable to guarantee 80% confluence during experiment completion. Twenty-four hours after plating, samples were added in the medium to the cells dissolved in sterile saline buffer and the respective negative controls were included in the experiment. FdU were dissolved in sterile 0.5% (*v*/*v*) DMSO in a culture medium and plated in the same range of concentrations used for the DNA oligonucleotides sequences. After 48 h of incubation at 37 °C under 5% CO_2_, the MTT reagent was added to a final concentration of 0.5 mg mL^−1^ to each well and incubated for 2 h. The MTT reduction was read at a single wavelength of 570 nm.

### 4.5. Apoptosis by Flow-Cytometry

The proportion of apoptotic cells resulting from exposition to the DNA oligonucleotides were evaluated by flow cytometry combining fluorescein isothiocyanate (FITC)-annexin V and PI. Cells were seeded in duplicate in 24-well plates with a density of 5 × 10^4^ cell well^−1^. Cells were treated with the modified FdU_5_ sequences and the controls and incubated for 48 h. The attached cells were harvested with trypsin, and floating cells were washed once in cold PBS, pelleted and resuspended in annexin binding buffer plus FITC-annexin V and PI according to the manufacturer’s specifications. After staining, samples were analyzed by flow cytometry.

### 4.6. Statistical Analysis

To estimate the inhibitory concentration (IC_50_) of each conjugate and its 95% confidence intervals, nonlinear regression models were used assuming a symmetrical sigmoidal four-parameters curve [48] for the relationship (GraphPad Prism version 9.0.2 for Windows 64-bit, GraphPad software, San Diego, CA, USA). The response was used in the log form (log10(dose)) rather than the dose itself. After convergence of the models, goodness-of-fit was checked by looking at the replicates test, the residuals, the covariance matrix of the estimated parameters, the dependence between parameters, and the determination coefficient (R^2^).

To compare mean conjugates at the same dose, all pairwise comparisons were performed between conjugates using the unequal variance unpaired *t*-test (Welch’s correction). To correct for multiple comparisons, the false discovery rate (FDR) approach was used, setting the maximum desired FDR at 5% (Q = 0.05). The method of FDR used was the Two-stage step-up method of Benjamini, Krieger and Yekutieli [49,50].

To compare means of a conjugate between all doses, pairwise comparisons were performed using the same previous statistical methodology

## 5. Conclusions

In this work, we investigated the biological effects of several enhancers into an oligonucleotide carrying five floxuridine units. First, we observed that all these molecules could increase the internalization efficiency of the oligonucleotide conjugates with different degrees depending on the cell line. Interestingly, the presence of the palmitic acid moiety provided a higher increase in the cytotoxic effects of FdU oligomer with an IC_50_ value in the low micromolar range 1.4 µM in HCC2998, 279 nM in HTB-38 and 165 nM in HeLa. On the contrary, the cholesterol conjugate that was the best-internalized conjugate had poor antiproliferative activity, even worse than the unmodified floxuridine oligomers. As a result, we can conclude that incorporating palmitic and, to a lesser extent, folic acid has a clear benefit in the therapeutic action of the FdU oligomer that can also be extended for delivering other antiproliferative oligonucleotides.

## Figures and Tables

**Table 1:** HPLC analysis of the degradation of FdU_5_ and FdU_5_ conjugates by snake venom phosphodiesterase I (SVP) and bovine spleen phosphodiesterase II (BSP).

Oligonucleotide	Phosphodiesterase I (SVP)			Phosphodiesterase II (BSP)		
	1.5 h	4 h	24 h	1.5 h	4 h	24 h
(FdU)_5_	100%	-	-	100%	-	-
(FdU)_5_–**Chol**	<5% *	<5% *	<5% *	95%	100%	-
(FdU)_5_–**Pal**	5%	10%	75%	90%	100%	-
(FdU)_5_–**GalNac**	4%	8%	19%	8%	13%	21%
(FdU)_5_–**PEG**	95%	100%	-	30%	60%	100%
**Folic**–(FdU)_5_	70%	100%	-	10%	15%	30%

* In this case, the peak corresponding to chol–FdU_5_ was not observed, but the amount of FdU obtained was estimated by comparison with the amount observed in the degradation of the other conjugates.

**Table 2:** IC_50_ value of FdU_5_ and FdU_5_ conjugates.

Oligos	Fibroblast	HCC2998	HepG2	HeLa	HTB38
	CC_50_ (µM)	IC_50_ (µM)	IC_50_ (µM)	IC_50_ (nM)	IC_50_ (nM)
(FdU)_5_	>10,000	16.9 (10.0–33.1)	7.8 (5.6–11.3)	204 (157–265)	1150 (763–1810)
(FdU)_5_**–Chol**	>10,000	457 (153–2.2 mM)	>50	554 (382–823)	1650 (1108–2590)
(FdU)_5_**–Pal**	>100	1.35 (1.05–1.8)	9.3 (7.2–12.7)	165 (131–206)	279 (220–356)
(FdU)_5_**–GalNac**	>1300	36.5 (19.9–80.7)	>50	1140 (874–1490)	15,800 (10,800–24,900)
(FdU)_5_**–PEG**	>180	>50	65.8 (31–201)	309 (242–395)	1680 (1240–2230)
**Folic–**(FdU)_5_	>10,000	12.5 (9.05–18.4)	>50	206 (158–267)	870 (652–1170)

The confidence interval is shown between brackets.

## Data Availability

The data presented in this study are available on request from the corresponding authors.

## Supplementary Materials

The following are available online at https://www.mdpi.com/article/10.3390/ijms22115678/s1, Materials and methods; Preparation of the solid support functionalized with polyethyleneglycol.
