# Supplementary material for: Evaluation of Floxuridine Oligonucleotide Conjugates Carrying Potential Enhancers of Cellular Uptake
Source: Int J Mol Sci. 2021 May 26;22(11):5678. doi: 10.3390/ijms22115678 (PMC8199350; doi:10.3390/ijms22115678)
Supplement: Supplementary file 1 [file ijms-22-05678-s001.zip › ijms-1187115-supplementary.pdf]

Supplementary Material

Evaluation of floxuridine oligonucleotide conjugates carrying potential enhancers of cellular uptake

Anna Aviñó [1,2,*], Anna Clua [1,2], Mª José Bleda [1], Ramon Eritja [1,2,*] and Carme Fàbrega [1,2]

[1] Institute for Advanced Chemistry of Catalonia (IQAC-CSIC), Jordi Girona 18-26, E-08034 Barcelona, Spain
[2] Networking Center on Bioengineering, Biomaterials and Nanomedicine (CIBER-BBN)

* Correspondence: aaagma@cid.csic.es (A.A.); recgma@cid.csic.es (R.E.); Tel.: (+34-93-4006100)

Table of contents:

1. Materials and methods — Page 2
2. Synthesis of the solid support functionalized with polyethyleneglycol — Page 2
3. **Table S1**. List of Maldi-TOFs, HPLC retention times and mass spectra of the synthetized oligonucleotides. — Page 3
4. **Figure S1.** Enzymatic degradation of FdU_5 and the FdU_5 conjugates — Page 5
5. **Figure S2.** Intracellular uptake of FdU_5 and FdU_5-conjugates at 5 µM — Page 6
6. **Figure S3**. MTT cell viability assay of FdU pentamer FdU_5 in the four cancer cell lines and healthy fibroblast cells. — Page 7
7. **Figure S4.** MTT significance in the cell viability assay of FdU pentamer FdU_5 and FdU_5 conjugates with the different carriers in HCC2998 cells. — Page 8
8. **Figure S5.** MTT significance in the cell viability assay of FdU pentamer FdU_5 and FdU_5 conjugates with the different carriers in HTB38 cells. — Page 9
9. **Figure S6.** MTT significance in the cell viability assay of FdU pentamer FdU_5 and FdU_5 conjugates with the different carriers in HeLa cells. — Page 10
10. **Figure S7.** MTT significance in the cell viability assay of FdU pentamer FdU_5 and FdU_5 conjugate with the different carriers in HepG2 cells. — Page 11
11. **Figure S8.** MTT significance in the cell viability assay of FdU pentamer FdU_5 and FdU_5 conjugates with the different carriers in Fibroblast cells. — Page 12

1. *Material and methods*

1.1. Reagents: The standard phosphoramidites and ancillary reagents used on the oligonucleotide synthesis were obtained from Applied Biosystems and LGC Link Technologies (Lanarkshire, Scotland, UK). 5'-Fluorescein CE phosphoramidite (FITC), 5-FdU-CE phosphoramidite and amino-modified-C6 linker were acquired from LGC Link Technologies or Glen Research (Sterling, Virginia, USA). 5-Fluoro-2'-deoxyuridine (FdU) was purchased from Alfa Aesar (Thermo Fisher, Kandel, Germany). The solid supports functionalized with cholesterol and palmitic acid were commercially available from LGC Link Technologies (Lanarkshire, Scotland, UK) and GalNAc solid support from Primetech ALC (Minsk, Belarus). Matrix for MALDI-TOF experiments was composed by 2',4',6'-trihydroxiacetophenone monohydrate (THAP, Aldrich, Madrid, Spain) and ammonium citrate dibasic (Fluka, Madrid, Spain). Solvents for HPLC analysis were prepared using triethylammonium acetate (TEAA) and acetonitrile (Merck, Madrid, Spain) as mobile phase. The desalted columns with Sephadex G-25 (NAP-10 were from GE Healthcare (Little Chalfont, UK). The rest of the chemicals are analytical reagent grade from commercial sources as specified. Ultrapure water (Millipore) was used in all experiments. HTB-38, HeLa and HepG2 cell lines were purchased from American Type Culture Collection, Manassas, VA. HCC2998 cell line was kindly provided by Dr. Diego Arango (Molecular Oncology Group; CIBBIM-Nanomedicine, Vall d'Hebron Institut of Research (VHIR) Barcelona, Spain). Healthy primary fibroblast cells were cultured from a healthy donor and were kindly provided by Drs. Fina Cases and Gemma Fabrias (IQAC-CSIC). Cell lines were cultured in Nunc® 10cm culture dishes (Thermo Scientific, Waltham, MA, USA) and were grown in DMEM supplemented with 10% fetal bovine serum (FBS). Cultures were grown at 37 °C in 5% CO_2 humidified atmosphere. DMEM (Dulbecco's Modified Eagle Medium), PBS (phosphate buffered saline containing disodium hydrogen phosphate, sodium chloride and potassium chloride) and RNAse free water were purchased from Gibco (Waltham, Massachusetts, USA). Annexin V-FITC kit was obtained from ThermoFisher (Waltham, Massachusetts, USA) and used following manufacturer's instructions.

1.2. Instrumentation: Modified oligonucleotides were synthesized on an ABI 3400 DNA Synthesizer (Applied Biosystems, Foster City, CA. USA). Semipreparative RP-HPLC was performed on a Waters chromatography system (Milford, Massachusetts, USA) with a 2695 Separations Module equipped with a Waters 2998 Photodiode Array Detector using Nucleosil 120 C18 (250x8mm) column. Mass spectra were recorded on a MALDI Voyager DETM RP time-of-flight (TOF) spectrometer (Applied Biosystems, Foster City, CA. USA). Molecular absorption spectra between 220 and 550 nm were recorded with a Jasco V650 spectrophotometer. The temperature was controlled with an 89090A Agilent (Santa Clara, CA, USA) Peltier device. Hellma (Jena, Germany) quartz cuvettes were used. Gels were imaged with a Gene Genius Bioimaging system (Syngene International Ltd. Bangalore, India). The MTT and colony assays were measured in an automated spectrophotometric plate reader Glomax multi detection system (Promega, Madison, WI, USA). Internalization and apoptosis assays were measured with a Guava easyCyte™ flow cytometer (Millipore, Burlington, MA, USA) and data were analyzed with Guavasoft 3.1.1.

2. *Synthesis of the solid support functionalized with polyethyleneglycol*

The synthesis of the solid support functionalized with polyethyleneglycol has been done as described in ref. [29] with small modifications.

2.1. Synthesis of 4, 4'-dimethoxytrityl-polyethyleneglycol 1000

A solution of 450 mg (0.71 mmol) of polyethyleneglycol 1000 (ABCR GmbH, Karlsruhe, Germany) was dried three times by coevaporation with anhydrous pyridine (3x5ml) and finally dissolved in 5 ml of anhydrous pyridine under nitrogen atmosphere. Then, 150 mg (0.44 mmol) of 4,4′-dimethoxytriphenylmethyl chloride (DMT-Cl). After 2 h of reaction, 1 ml of MeOH was added to quench the reaction and the solvent was eliminated by evaporation. The crude of the reaction was purified by column chromatography over silica gel eluted with a gradient of 0-5% of MeOH in DCM with 1% of Et_3N.

^{1}H-NMR ($CDCl_3$): δ 7.4–7.20 (m, 9H, DMT), 6.7 (d, 4 H, DMT), 3.7 (s, 6H), 3.5 (s, nH) $(CH_2CH_2O)_n$ (PEG), 3.20 (t, 2H, (PEG) CH_2–O–DMT) ppm

2.2. Preparation of 5'-O-(4, 4'-dimethoxytrityl)-polyethyleneglycol 3'-O-succinate

A solution of 70 mg (0.05 mmol) of the previous compound was dried by evaporation with anhydrous acetonitrile (ACN) under reduced pressure and finally dissolved with 6 ml of anhydrous dichloromethane (DCM). Then, 8 mg (0.08 mmol) of succinic anhydride and 9.7 mg (0.08 mmol) of 4-(dimethylamino)pyridine were added. The reaction mixture was stirred at room temperature (r.t.) overnight. The mixture was diluted with DCM and washed with 0.1 M NaH_2PO_4 pH 5 and saturated aqueous NaCl. The organic phase was dried with Na_2SO_4, filtrated and evaporated to dryness. The resulting DMT-PEG hemisuccinate compound was obtained as a white solid and used in the next step without further purification.

2.3. Synthesis of the solid support containing the PEG derivative.

The DMT-PEG hemisuccinate derivative was incorporated to a long-chain alkylamine-controlled pore glass support (LCAA-CPG) as described (Gupta KC et al., Nucleosides & Nucleotides 1995, 14, 829-832). Briefly, 20 mg (0.02 mmol) of the DMT-PEG hemisuccinate and 3.6 mg (0.03 mmol) of DMAP were dissolved in 500 μL of acetonitrile. To this solution, 9.3 mg (0.03 mmol) of 2,2'-dithio-bis-(5-nitropyridine) dissolved in 120 μL of a mixture of ACN:DCM (1:3) were added. Then, a solution containing 7.8 mg (0.03 mmol) of triphenylphosphine in 60 μL of acetonitrile was added. The reaction mixture was stirred for seconds and poured to 200 mg of LCAA-CPG (70 μmol/g) that had been previously washed with methanol (MeOH), DCM and acetonitrile. The mixture was left to react for 4 h. The support was washed with MeOH, DCM and ACN and dried under vacuum. Finally, the DMT-PEG support was treated with a mixture of capping mixtures employed in the DNA synthetizer for 10 min to cap unreacted amino groups.

Table S1. List of Maldi-TOF and HPLC retention time (Rt) of the synthetized oligonucleotides

Sequences (5'-3')	M Expected	M Found	Rt (min)
$(FdU)_5$	1479	1477	12.4
$(FdU)_5$**-Chol**	2172	2179	22.0
$(FdU)_5$**-Pal**	2014	2010	17.2
$(FdU)_5$**-GalNac**	3266	3265	12.2
$(FdU)_5$**-PEG**	~2493	Broad ~2482	8.1
**Folic-**$(FdU)_5$	2083	2079	15.8

HPLC conditions: Nucleosil 120–10 C18 column (250 × 4 mm). Solvent A: 5% ACN in 0.1 M aqueous TEAAc (pH = 7) and solvent B: 70% ACN in 0.1 M aqueous TEAA (pH = 7). Flow rate: 1 mL/min. Conditions: 20 min linear gradient from 0% to 50% B for $(FdU)_5$ and GalNAc, PEG and Fol derivatives and from 15% to 100 % B in 30 for Pal and Chol derivatives.

Maldi-TOF spectra of $(FdU)_5$, $(FdU)_5$-Chol, $(FdU)_5$-Pal, $(FdU)_5$-GalNAc, $(FdU)_5$-PEG, Folic-$(FdU)_5$

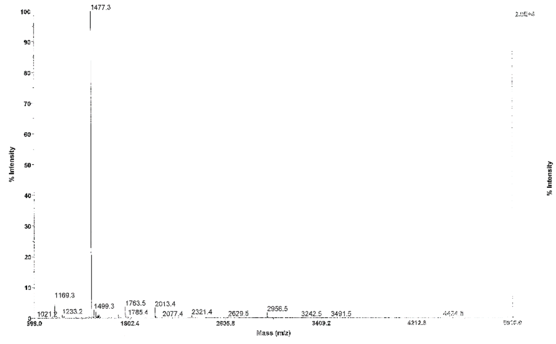

$(FdU)_5$

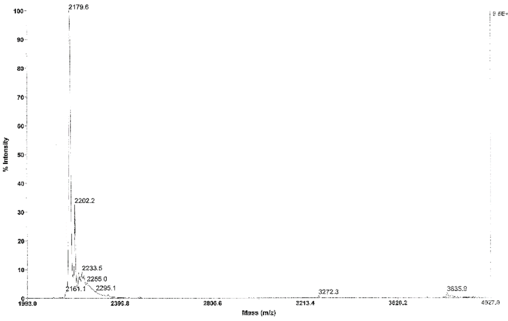

$(FdU)_5$-Chol

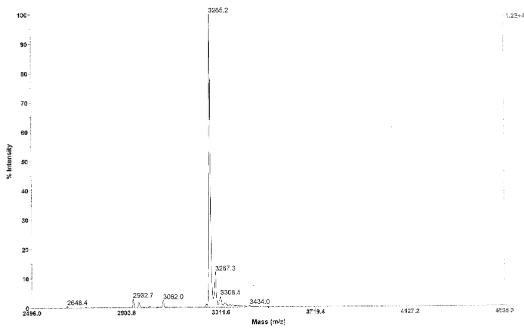

$(FdU)_5$-GalNAc

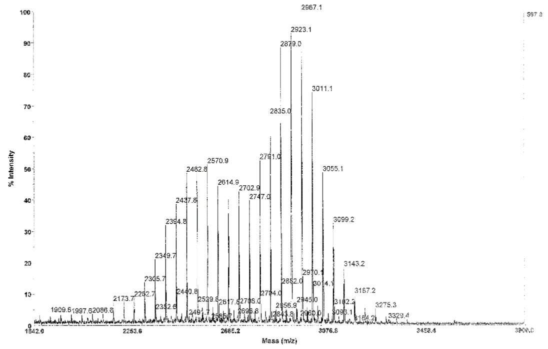

$(FdU)_5$-PEG

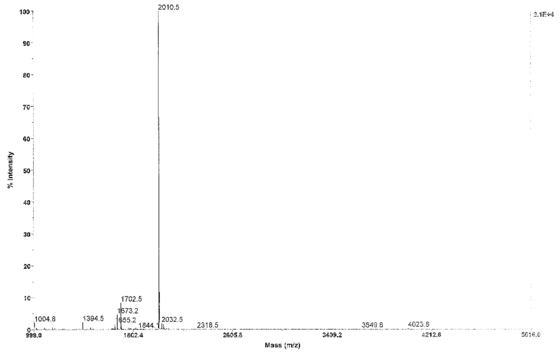

Folic-$(FdU)_5$

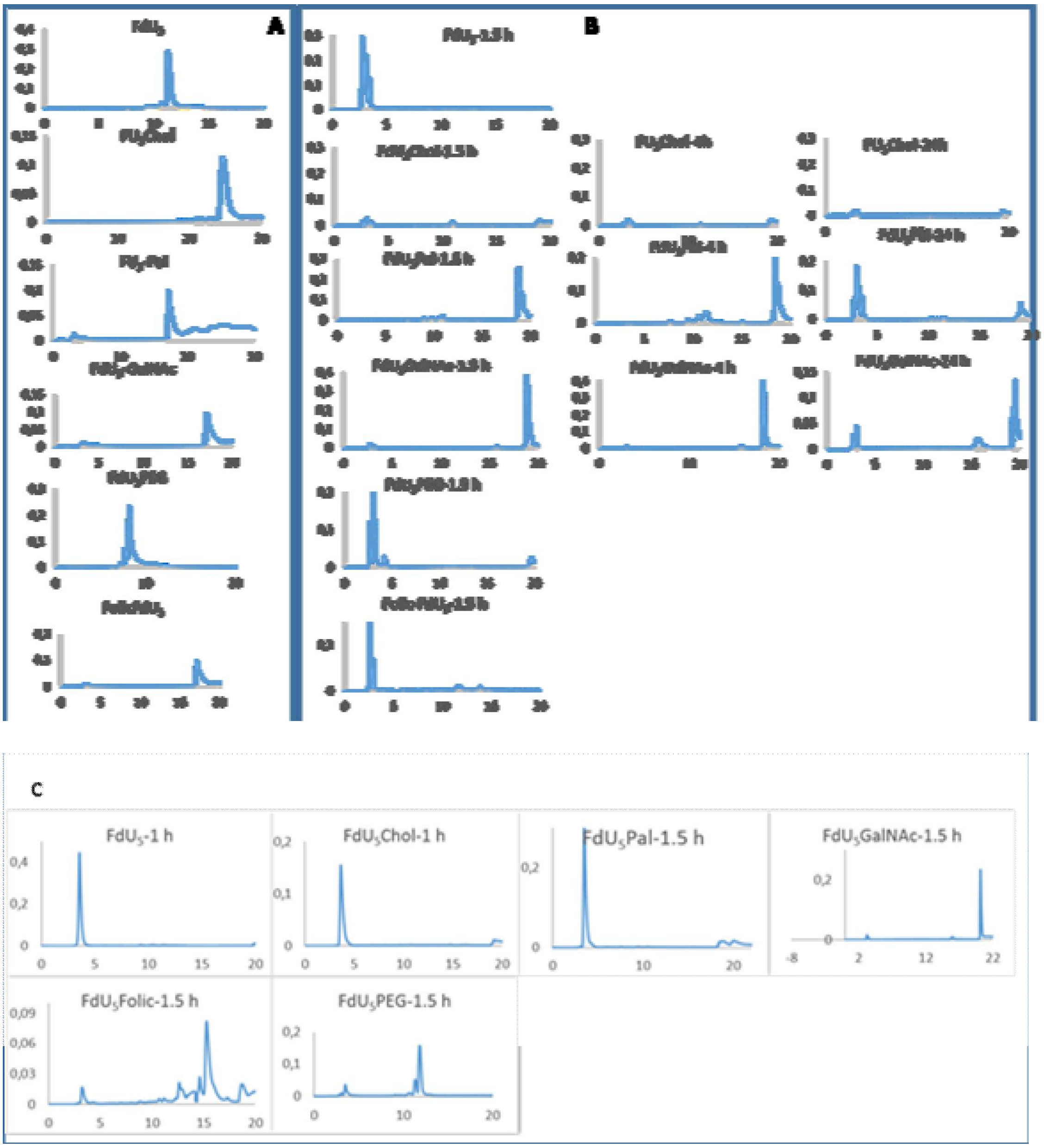

Figure S1. HPLC analysis of FdU_5 and FdU_5-conjugates (A), the enzymatic digestions with snake venom phosphodiesterase I (B) and the enzymatic digestions with bovine spleen phosphodiesterase II (C). The HPLC analysis conditions used in A are as follows: a 20 min linear gradient from 0 to 50 % B, except for FdU_5-Pal and FdU_5-Chol that a 20 min linear gradient from 15 to 85 % with 10 min at 100% B was used. Column: Nucleosil C18, 10μm, 250x4 mm. Buffer A: 5% ACN 0.1 M TEAAc and Buffer B: 70% ACN 0.1 M TEAAc. HPLC analysis of the digestion mixture (B and C) was done using a 15 min linear gradient from 0 to 25 % B to analyze the degradation products and then 5 min at 100% B to elute the non-degradated oligonucleotides.

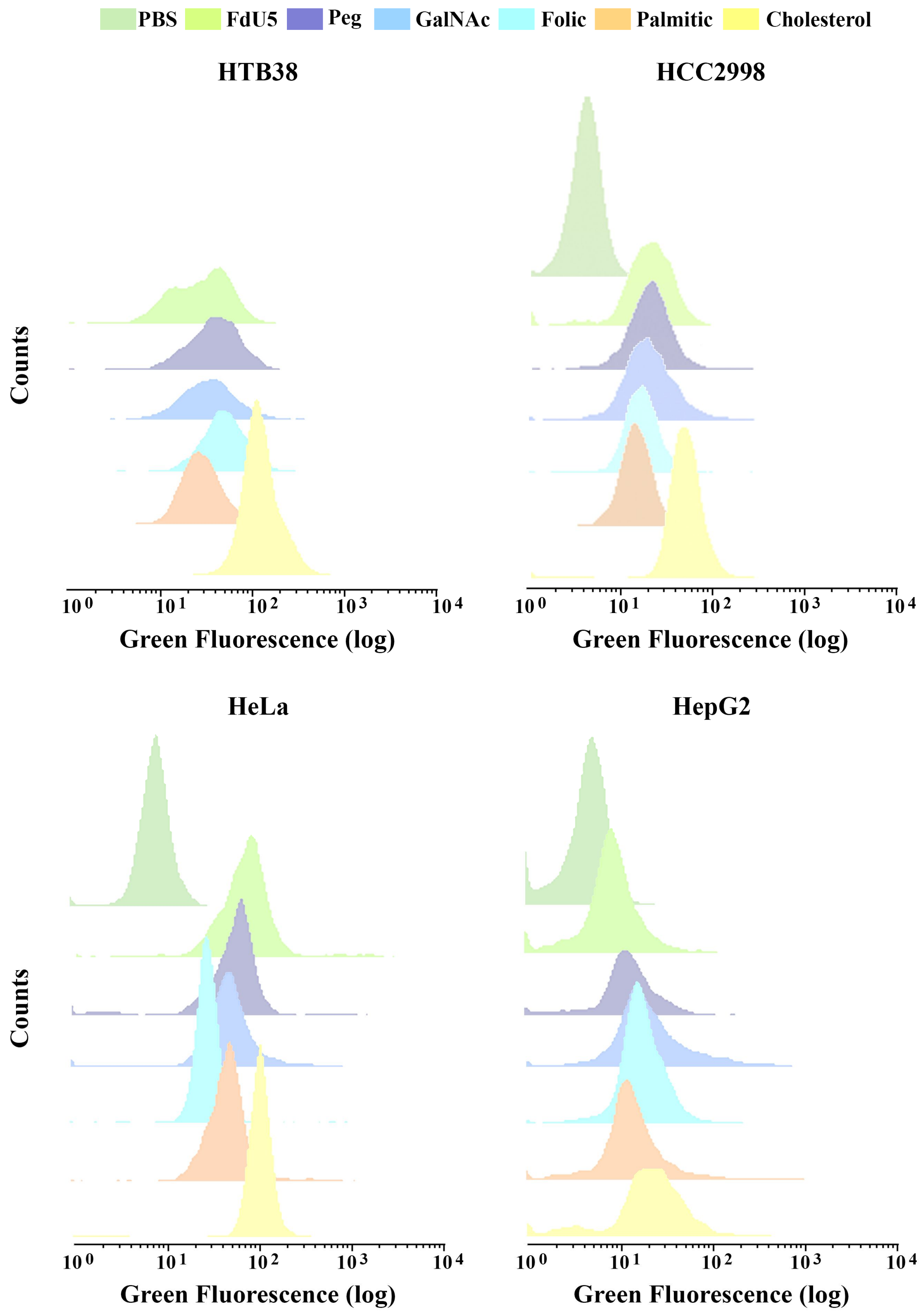

Figure S2. Intracellular uptake of FdU_5 and FdU_5-conjugates molecules in HTB38, HCC2998, HeLa and HepG2 cell lines. Fluoresceine labelled FdU_5 and FdU_5-conjugates at 5 µM were incubated separately with the cells and the internalization is shown as obtained by flow cytometry.

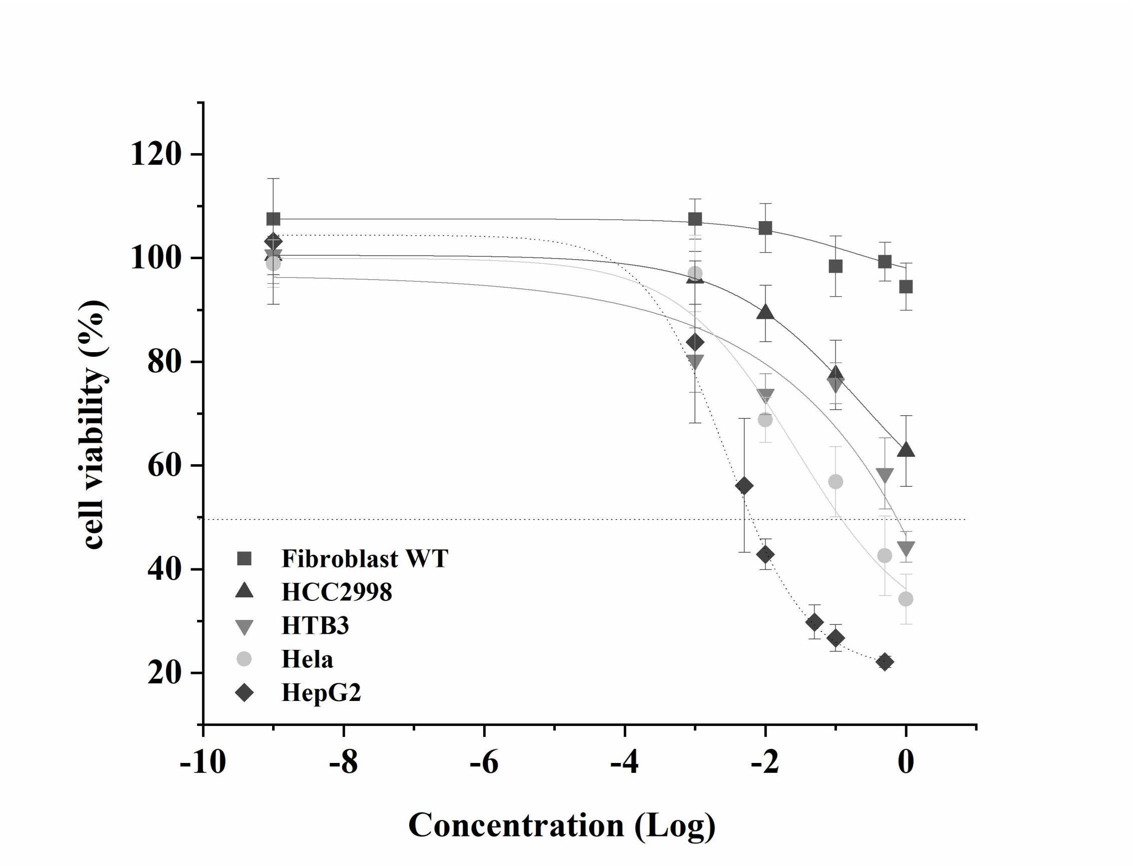

Figure S3. MTT cell viability assay of FdU pentamer FdU_5 in the four cancer cell lines and the healthy fibroblast cells. The concentration was assayed range from 1 nanomolar (nM) to 10 micromolar (µM). The values for $(FdU)_5$ are shown as fill black square (Fibroblast WT), fill dark grey triangles (HCC2998), fill medium grey inverted triangles (HTB38), fill light grey cycles (HeLa) and fill dark grey triangles (HepG2). Error bars represent the standard deviation (SD) of two independent experiments in triplicate.

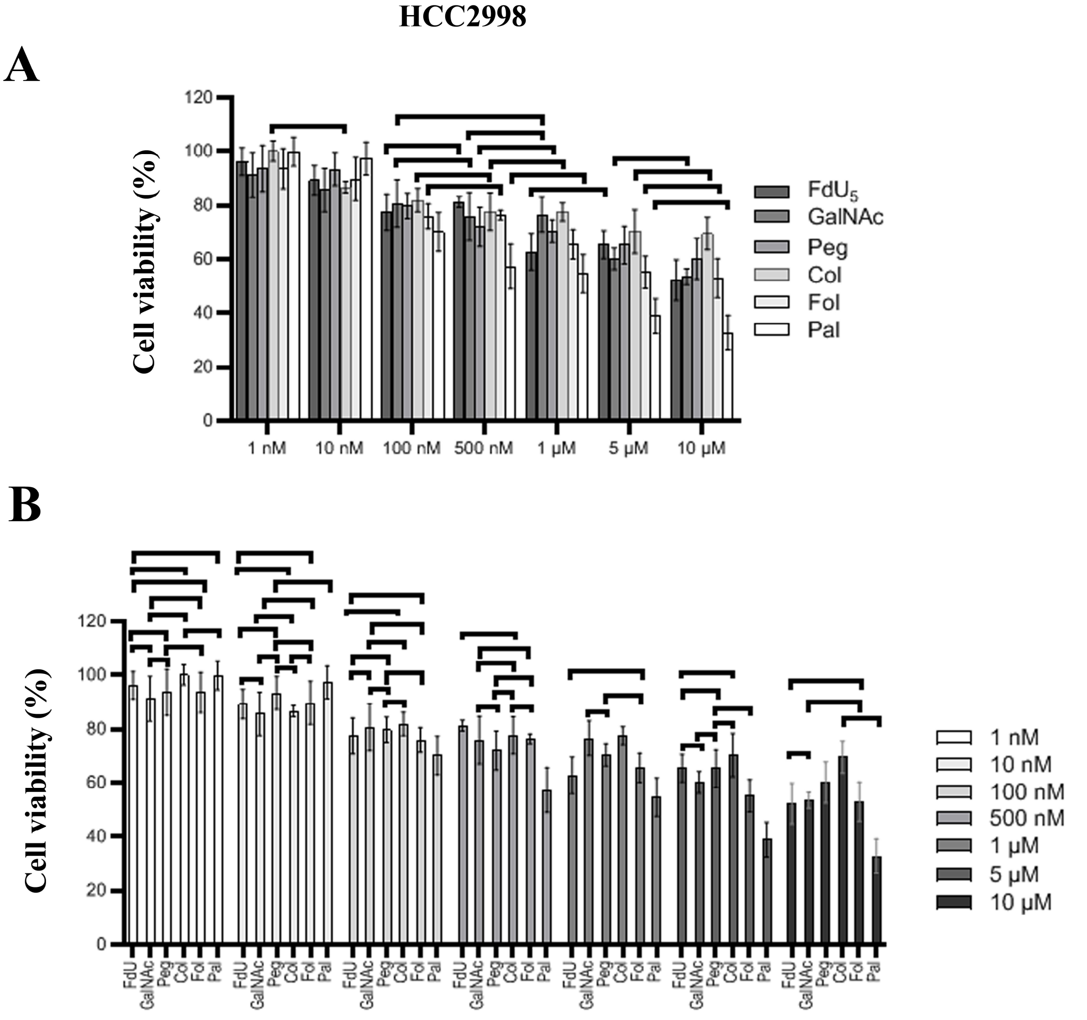

Figure S4. MTT significance in the cell viability assay of FdU pentamer FdU_5 and FdU_5 conjugates with the different carriers in HCC2998. The bars represent the pair with no statistical significance.

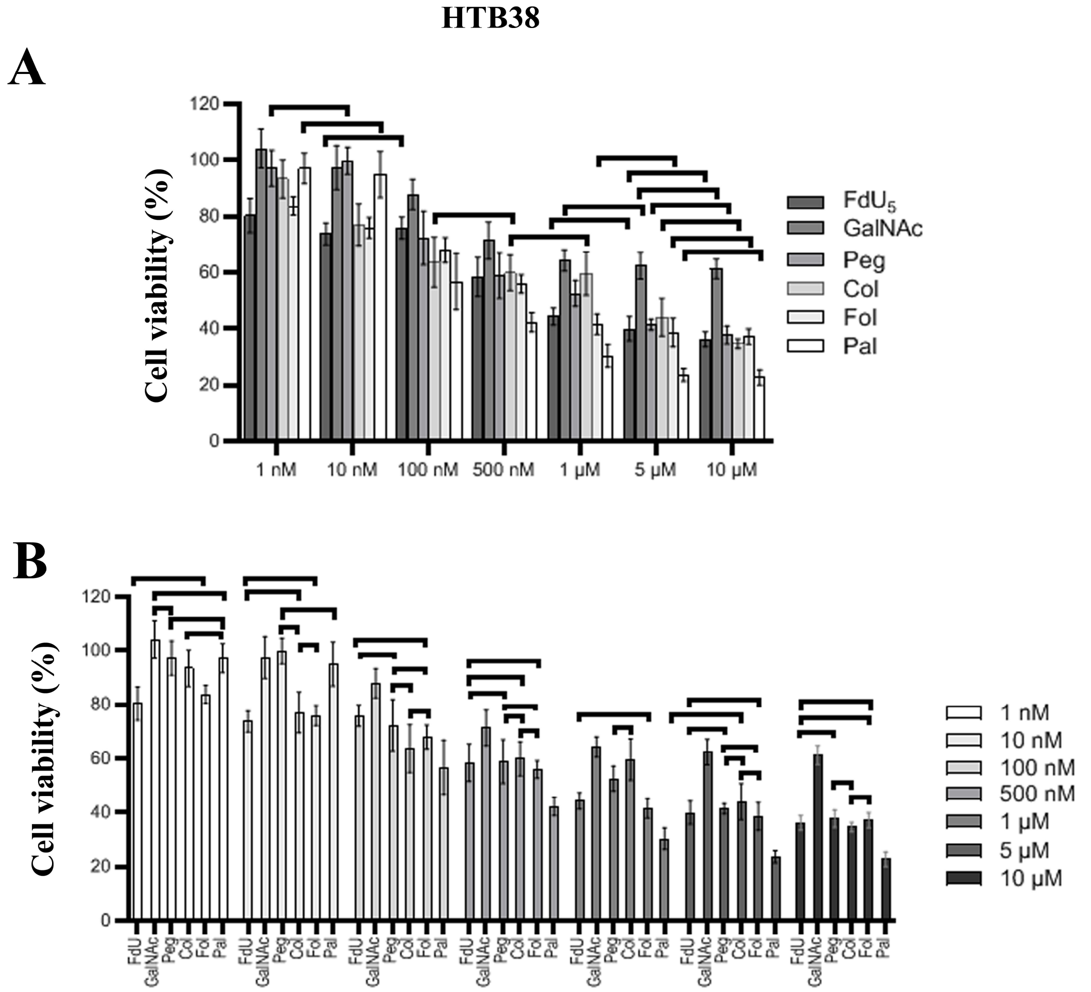

Figure S5. MTT cell viability assay of FdU pentamer FdU_5 and FdU_5 conjugates with the different carriers in HTB38 cells. The bars represent the pair with no statistical significance.

HeLa

A

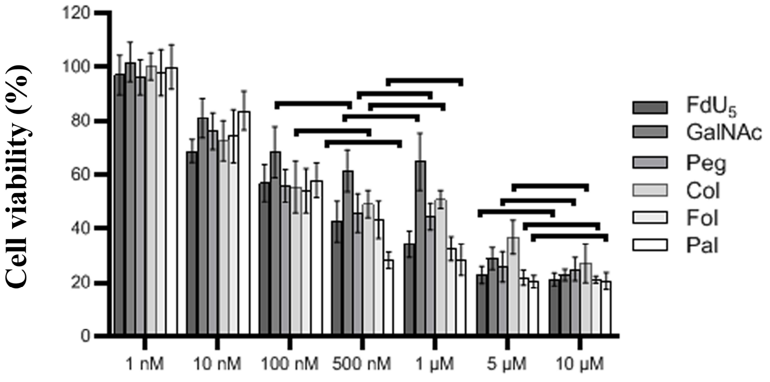

B

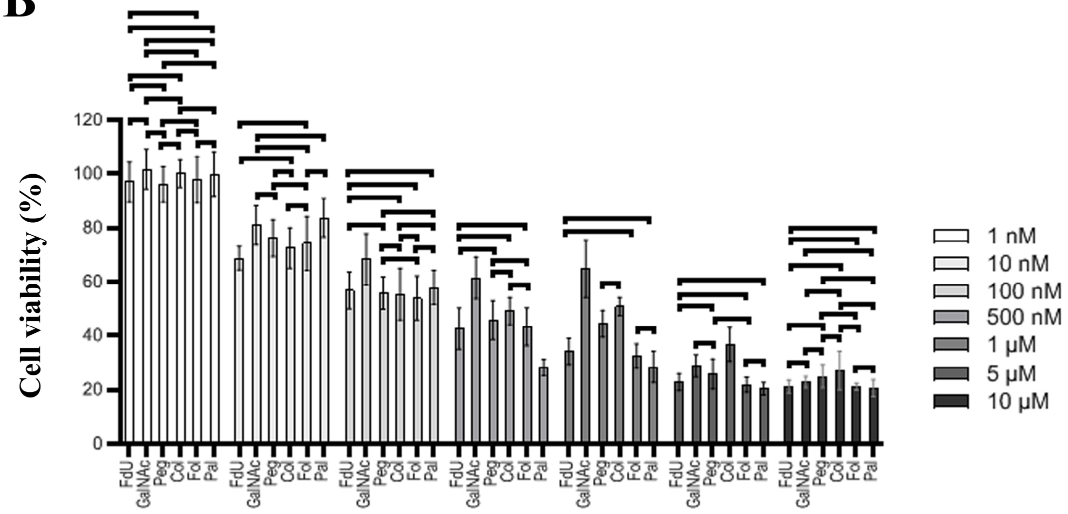

Figure S6. MTT cell viability assay of FdU pentamer FdU_5 and FdU_5 conjugates with the different carriers in HeLa. The bars represent the pair with no statistical significance.

HepG2

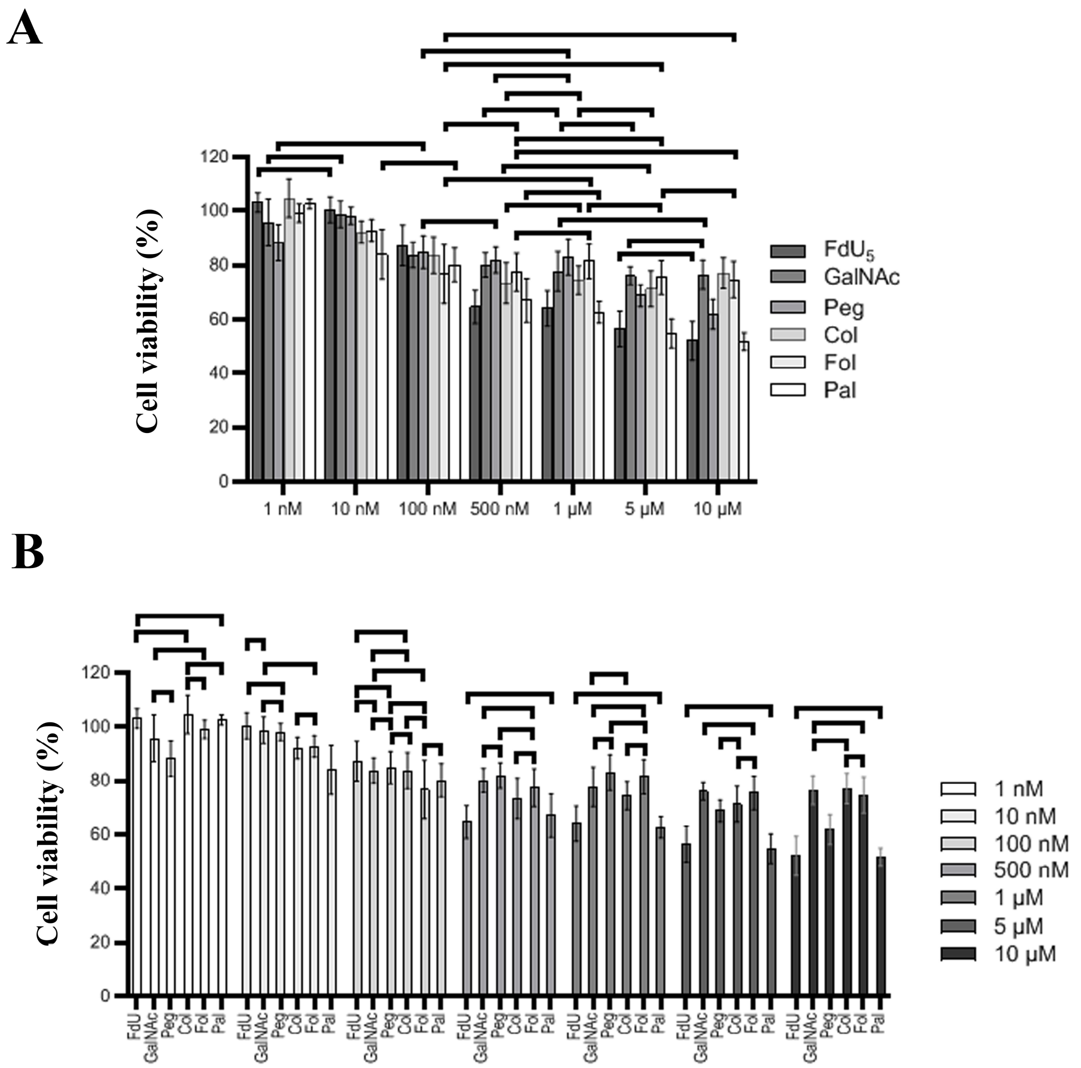

Figure S7. MTT cell viability assay of FdU pentamer FdU_5 and FdU_5 conjugates with the different carriers in HepG2. The bars represent the pair with no statistical significance.

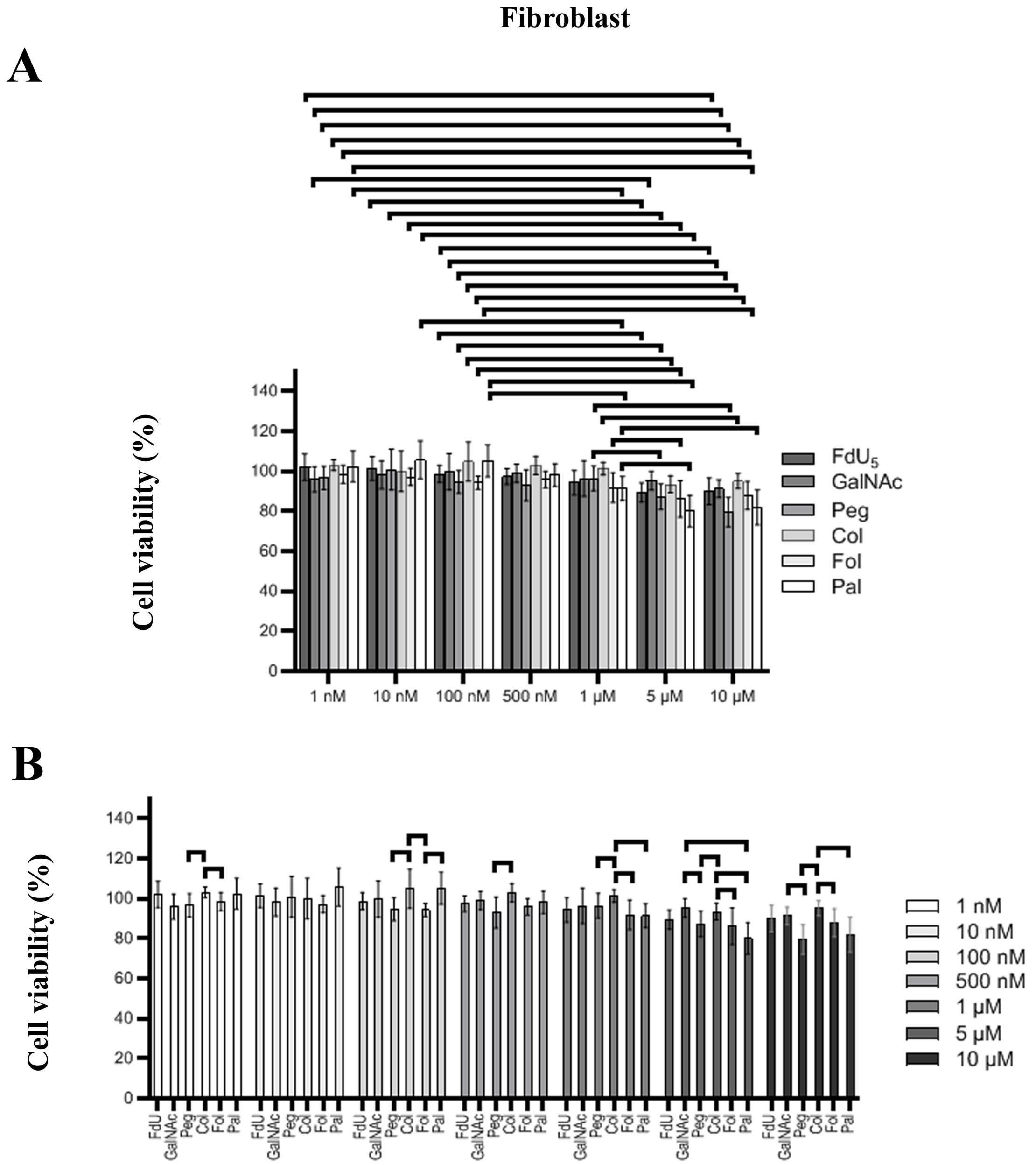

Figure S8. MTT cell viability assay of FdU pentamer FdU_5 and FdU_5 conjugate with the different carriers in Fibroblast cells. The bars represent the pair with statistical significance.
